# The State of Behavior Change Techniques in Virtual Reality Rehabilitation of Neurologic Populations

**DOI:** 10.3389/fpsyg.2019.00979

**Published:** 2019-05-08

**Authors:** Danielle T. Felsberg, Jaclyn P. Maher, Christopher K. Rhea

**Affiliations:** ^1^Virtual Environment for Assessment and Rehabilitation (VEAR) Lab, Department of Kinesiology, University of North Carolina at Greensboro, Greensboro, NC, United States; ^2^Physical Activity and Lifetime Wellness Lab, Department of Kinesiology, University of North Carolina at Greensboro, Greensboro, NC, United States

**Keywords:** virtual reality, neurorehabilitation, gait, mobility, motor performance, behavior change techniques

## Abstract

**Background:** Neurologic rehabilitation aims to restore function, address barriers to activity, and improve quality of life in those with injury to the nervous system. Virtual reality (VR) has emerged as a useful tool to enhance neurorehabilitation interventions and outcomes. However, the manner in which VR-based neurorehabilitation has been manipulated to optimize outcomes using theory-based frameworks has not been documented. Behavior Change Techniques (BCTs) are described as the smallest active ingredient in an intervention aimed to change behavior via theoretically-proposed pathways. The purpose of this review was to investigate the ways VR is being used in neurorehabilitation to improve upright mobility, and systematically code those VR interventions for active BCTs.

**Methods:** Keyword searches were performed using database searches of PubMed, SPORTDiscus, and psycINFO. The search yielded 32 studies for inclusion. Coding for BCTs was conducted using the Behavior Change Techniques Taxonomy v1 (BCTTv1).

**Results:** Behavioral Practice, Graded Tasks, Biofeedback, and Explicit Feedback were the most commonly used BCTs. All studies reported improvements in motor performance outcomes. However, none of the studies investigated the efficacy of each component of their VR intervention making it difficult to point to the most effective components of VR interventions overall.

**Conclusions:** This review suggests that investigation into the specific components of VR interventions, along with purposeful implementation and reporting of BCTs will help improve understanding of the efficacy of VR as a neurorehabilitation tool. Future research could benefit from incorporating BCTs into the design process of VR interventions to produce optimal rehabilitation potential.

## Introduction

### Rationale

Virtual reality (VR) is an artificial environment containing sensory information (typically in the visual and/or auditory domains) that allows a natural expression of behaviors to emerge (Steinicke et al., 2013). There has been a wide range of contexts to which VR has been applied, including, but not limited to therapeutic programs addressing PTSD, anxiety, phobias, schizophrenia, ADHD, autism, and pain management (Freeman, 2008; Parsons and Rizzo, 2008; Li et al., 2011; Wang and Reid, 2011; Gonçalves et al., 2012; Opriş et al., 2012; Kandalaft et al., 2013). However, its usage in the physical rehabilitation of neurologic populations is relatively new and has grown in recent years (Darekar et al., 2015; Teo et al., 2016; Howard, 2017; Aida et al., 2018; Porras et al., 2018). VR in rehabilitation can take many forms, and is constantly evolving as the field and technology progresses (Kiefer et al., 2013). VR interventions can occur on a continuum of least (or non-) immersive to fully immersive. Changes in the components of the VR design can improve the level of immersion of the experience by the user (Patel et al., 2006). These components include: visual field continuity, interactivity, level and type of feedback, narrative engagement, user freedom, and visual conformity. With large developments in technology, the use of VR has increasingly emerged as a useful tool to enhance physical rehabilitation interventions and outcomes (Ravi et al., 2017; Casuso-Holgado et al., 2018; Porras et al., 2018).

Physical rehabilitation aims to restore function and improve quality of life in those with disability or injury. More specifically, neurologic physical rehabilitation is a specialized area of practice assessing and treating people with injury to the nervous system. For this population, barriers to physical activity include fear of falling, poor outcome expectations, and lack of social support (Mulligan et al., 2012; Ellis et al., 2013). Rehabilitation can help address these barriers and improve function, independence, and quality of life. VR has been shown to be an especially promising tool in rehabilitation to optimize mobility, as it allows patients to actively participate and interact in a dynamic environment (Keshner and Fung, 2017; LoJacono et al., 2017; Rhea and Kuznetsov, 2017; Porras et al., 2018). Furthermore, therapists can individualize VR interventions to best suit their intended outcomes and assess resultant behaviors.

In a recent systematic review and meta-analysis, VR interventions were shown to be more effective at improving motor outcomes than more traditional real-world interventions (Howard, 2017). Rehabilitation interventions, including gait training, used in combination with VR can provide an enriching and optimally stimulating environment (Molina et al., 2014). Zimmerli et al. (2013) outlined five critical components to create an ideally stimulating and optimally engaging VR task. These components are adaptability, explicit feedback, task goals, interactivity, and the option of added competition. For example, an adaptable VR task would be able to scale the difficulty of the task in real-time to adjust to the user's individual needs. Explicit feedback could be given through visual or auditory cues which allow the user to know if they are performing the task correctly, and task goals can be set at the beginning of the VR task. Interactivity speaks to the synchronicity of the user's behavior in the real-world to the outcome in the virtual world. A VR task with high interactivity could have an avatar, or similar representation of the user, which performs actions in the VR in concert with the user's movements in the real-world. Adding the option of competition to a VR would allow the user to perform the task in the VR while competing against a virtual opponent.

Currently, it is not clear the degree to which researchers are incorporating these five components in the design of VR interventions for motor rehabilitation. Moreover, while many of the aforementioned studies have used motor learning principles to design their VR rehabilitation program, adopting only principles of change in the motor domain may limit the adoption and effectiveness of the VR program. A more holistic approach would be to re-conceptualize VR rehabilitation programs in terms of a theoretically-informed framework that describes behavior change using a multi-domain perspective. One such framework applicable in this context is a taxonomy of Behavior Change Techniques (BCTs), which describes the “active ingredients” across a variety of domains needed to change behavior. A taxonomy linking theoretical constructs to BCT components was first developed by Abraham and Miche (2008) and subsequently updated to the “BCT Taxonomy v1” (Abraham and Miche, 2008; Michie et al., 2008, 2013). Although this taxonomy is relatively new, it has already been adopted across a variety of fields in which a change in behavior is desired (Webb et al., 2010; Bird et al., 2013; Conroy et al., 2014; Lyons et al., 2014). However, we are unaware of any papers that have applied this taxonomy of BCTs to examine components of neurologic rehabilitation in VR-based interventions. Systematically identifying BCTs could help characterize the components of past VR research that has led to the most desirable outcomes, providing a roadmap for future researchers and clinicians who wish to use VR as a tool to enhance physical rehabilitation.

### Objectives

The purpose of this review is two-fold: (1) to investigate the ways VR has been used in neurologic rehabilitation to improve motor performance and (2) to systematically code and analyze the state of BCTs in VR neurologic rehabilitation interventions.

### Research Question

The current state of VR interventions to promote improvements in gait and upright mobility in those with neurologic deficits is constantly evolving. Specifically, how current VR interventions are incorporating BCTs to create a more holistic intervention to address mobility deficits is unclear.

## Methods

### Study Design

This study was designed to be a systematic review of the current literature investigating the role of VR interventions to improve gait and upright mobility in participants with neurologic deficits across the lifespan. The articles selected for inclusion were coded using the Behavior Change Technique Taxonomy v1 (BCTTv1) by two independent coders. One coder was an expert in BCTs, and the other had expertise with VR interventions for motor rehabilitation. First, both coders independently categorized the initial thirty-percent of included articles, then met to come to a consensus on use of specific BCTs for final coding of all remaining articles. Disagreements between coders were resolved through discussion following independent coding of all articles.

### Participants, Interventions, Comparators

Articles were chosen for review if the study specifically investigated the effects of a VR intervention on an upright mobility-related outcome. Once studies were selected, all VR interventions were coded using the BCTTv1. The BCCTv1 was created to code BCTs and develop a common language to analyze and replicate interventions in both research and practical settings (Michie et al., 2013). It is a structured taxonomy of 93 BCTs and offers a reliable method for identifying and analyzing the BCT in an intervention (Michie et al., 2013). Due to the objective of this study, only the VR portion of the intervention of each study was coded using this taxonomy.

### Systematic Review Protocol

The systematic review protocol guidelines described by the Preferred Reporting Items for Systematic Reviews and Meta-analysis (PRISMA) were adopted and applied to this review (Moher et al., 2015). Keyword searches for two of the databases were initially performed from October 2017 through November 2017. An additional keyword search was conducted in a third database in October 2018. Articles were screened based on the specific inclusion and exclusion criteria outlined in the following data extraction section. Articles meeting the initial criteria were kept for further full-text assessment of eligibility. Through full-text assessment of each article, articles were excluded using extensive exclusion criteria to find articles for final qualitative synthesis.

### Search Strategy

**Figure 1:**
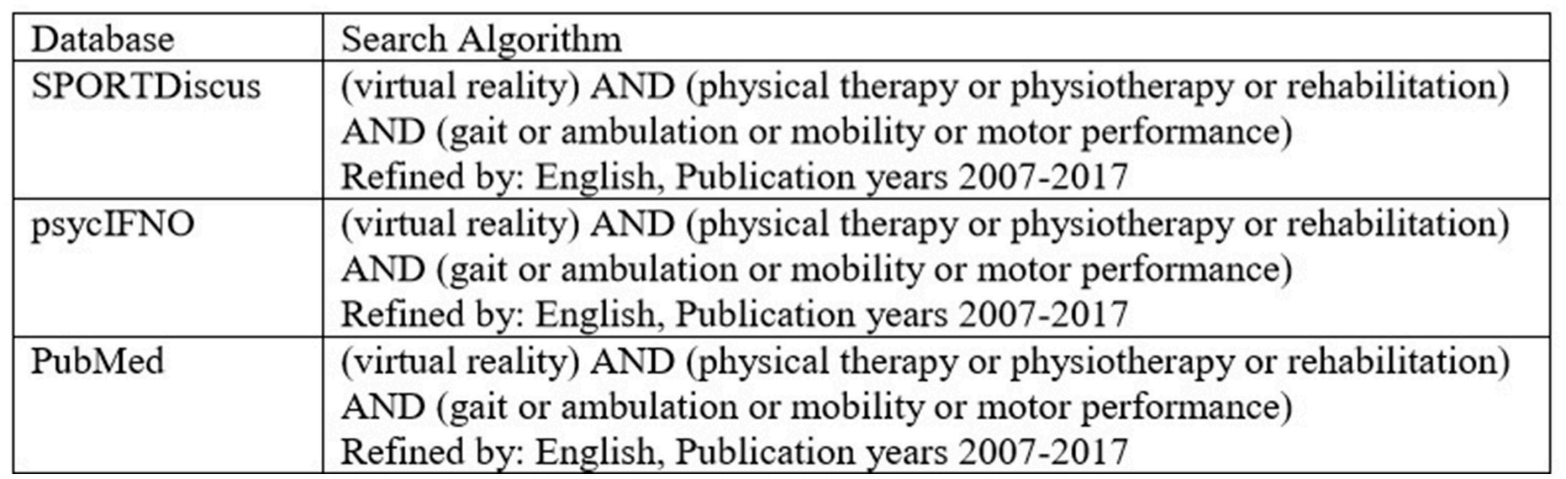
Database Search algorithm.

Keyword searches were performed using database searches of PubMed, SPORTDiscus, and psycINFO. The search parameters used included all possible combinations of (1) “virtual reality” (2) “physical therapy,” “physiotherapy,” or “rehabilitation” and (3) “gait,” “ambulation,” “mobility,” or “motor performance.” The specific search algorithm is provided in Figure 1.

### Study Selection

Once duplicates were removed, studies were excluded through the screening process if they were a systematic review or meta-analysis, a non-research text (editorial, commentary, etc.), did not have full-text available, or were a study protocol, pilot or case study. Additionally, studies were excluded if they did not use neurologic populations, an upright mobility task, or focused on the upper extremity. Following screening, all full-text articles were assessed for eligibility. Studies were selected for inclusion if they were investigating the role of VR in gait or upright mobility outcomes of neurologic populations. Studies were excluded if they did not use VR, were not directly assessing the relationship between VR and motor outcomes, used vestibular populations, or employed an adjunct therapy (i.e., transcranial magnetic stimulation).

### Data Sources, Studies Sections, and Data Extraction

Once the study selection process was completed, each article was reviewed to locate the section which detailed the VR intervention being used in the study. Only the section of the article regarding the VR intervention was used for BCT coding purposes. Other data (sample size, study design, and VR technology used, etc.) was extracted to synthesize the overall state of VR interventions aimed to address mobility deficits in neurologic populations by full-text review of the articles.

### Data Analysis

Following study selection and identification of the VR intervention sections of each included article, both coders independently coded these sections using the BCTTv1. Given the five components recommended in VR design of motor rehabilitation tasks (adaptability, feedback, task goals, interactivity and competition) (Zimmerli et al., 2013), it was expected that codes would be used from the following main taxonomy areas: goals and planning, feedback and monitoring, comparison of behavior, repetition and substitution, and antecedents, however coding was not restricted to these categories. The BCTs that are nested under these main taxonomies best align with the suggested VR design components identified by Zimmerli et al. (2013). Coders identified each BCT and gave the most appropriate corresponding code given definitions and descriptions in the BCTTv1. Prior to coding, both coders met to discuss standards for applying the BCTTv1 to VR task. This discussion was necessary to identify a systematic way in which to apply the BCTTv1 to the unique and dynamic nature of a VR task. After coding the first thirty-percent of the articles, both coders met and discussed areas of disagreement. Specifically, coders defined the use of antecedents as it applies to the virtual environment (VE) as this was the major area of disagreement. Following discussion, the coders agreed that the VR task would be given the code of “restructuring the physical environment” (taxonomy code: 12.1) if the VE had dynamic components that were added through the course of the VR task. Both coders also met after coding the remaining articles, again, to discuss areas of disagreement and establish a consensus regarding the BCTs being used in each VR intervention. Following this final meeting, a Cohen's Kappa statistic was calculated to demonstrate the level of agreement between coders.

## Results

### Study Selection and Characteristics

**Figure 2:**
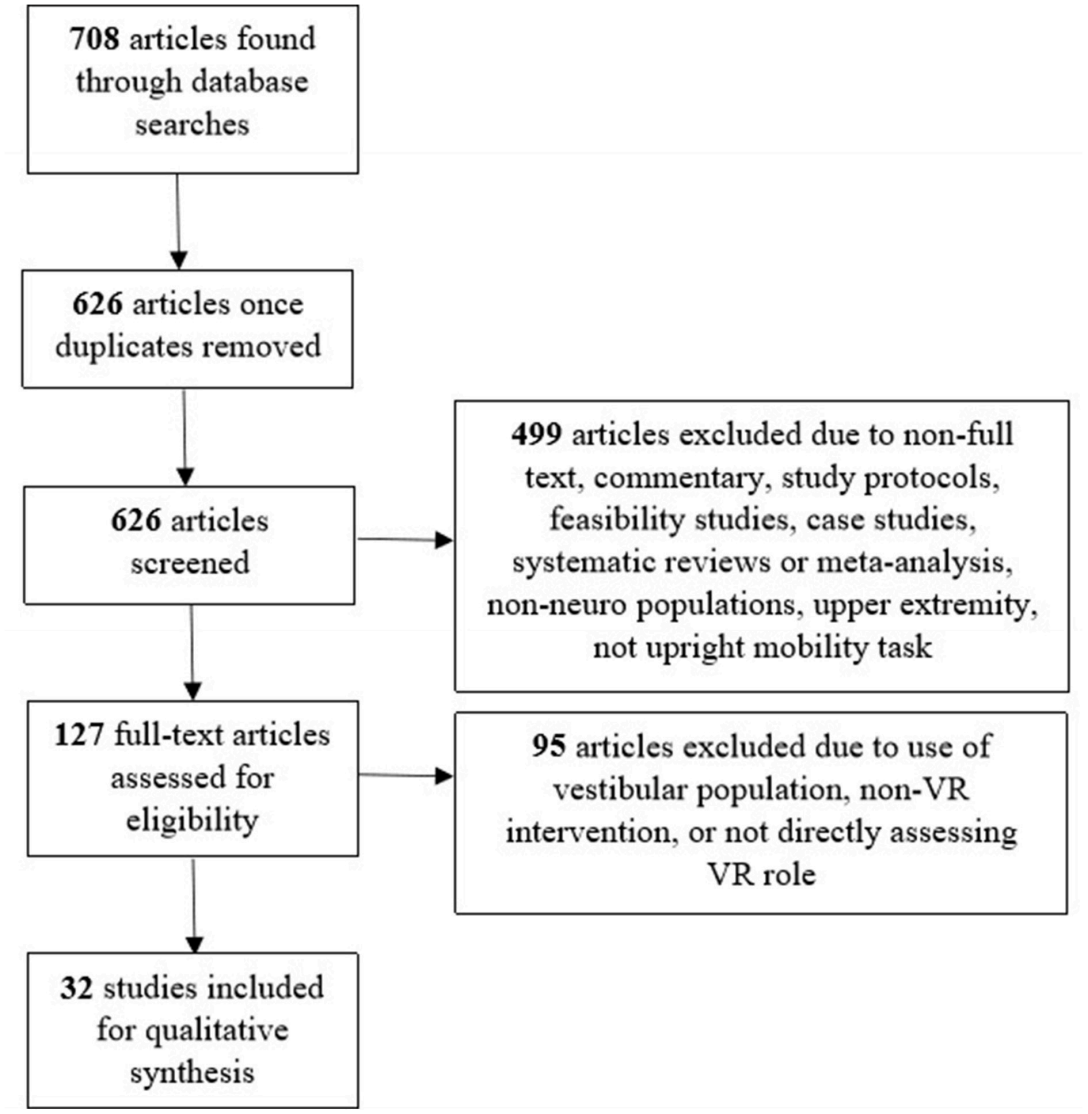
Flowchart of systematic article search and inclusion for review.

Figure 2 illustrates the article selection process. The initial searches of PubMed, SPORTDiscus, and psycINFO yielded 708 articles. Of these 708 articles, 82 were duplicates. After removal of duplicate articles, 626 articles were screened through titles and abstracts. Through the screening process, 155 articles were excluded because they were a systematic review, meta-analysis, case study, feasibility study, study protocol, non-full text or non-research text. Additionally, 344 articles were excluded because they did not use neurologic populations, focused on the upper extremity, or did not investigate an upright mobility task. Following the screening process, 127 full text articles were reviewed for eligibility. Of these 127 articles, 95 were excluded due to the following reasons: did not use a VR task, did not directly measure the role of VR on motor performance, or were vestibular populations. The 32 remaining articles were included for review (Lamontagne et al., 2007; Yang et al., 2008; Kim et al., 2009; Mirelman et al., 2009, 2010, 2011; Brien and Sveistrup, 2011; Brütsch et al., 2011; Schuler et al., 2011; Salem et al., 2012; Park et al., 2013; Singh et al., 2013; Villiger et al., 2013, 2017; McEwen et al., 2014; Cho et al., 2015, 2016; Killane et al., 2015; Lee, 2015; Liao et al., 2015; Song and Park, 2015; Wall et al., 2015; Yom et al., 2015; Bang et al., 2016; In et al., 2016; Biffi et al., 2017; Bonney et al., 2017; Calabrò et al., 2017; Gandolfi et al., 2017; Pedreira da Fonseca et al., 2017; Peruzzi et al., 2017; van Gelder et al., 2017).

### Synthesized Findings

#### Demographic Information

As per the inclusion criteria for this review, all 32 studies had subjects with neurologic diagnoses (Table 1). Thirty of the articles were conducted in a single neurologic population including stroke (*n* = 15), Parkinson's disease (*n* = 4), spinal cord injury (*n* = 3), and cerebral palsy (*n* = 3), multiple sclerosis (*n* = 2), brain injury (*n* = 1), developmental delay (*n* = 1), and developmental coordination disorder (*n* = 1). The remaining two articles used mixed clinical populations; one had adults with stroke, spinal cord injury, or brain injury; the other used a pediatric population of cerebral palsy, transverse myelitis, multiple sclerosis or brain injury. Twenty-four articles used adult subjects. Eight articles used pediatric participants. Sample sizes ranged from 4 to 76 total participants. The majority of studies (*n* = 14) had sample sizes in the twenties. Regarding the study design, 21 studies were randomized control study designs. Six used a clinical group with repeated measures. Moreover, 17 studies conducted at least part of the study in a controlled research laboratory, 12 were in clinical settings, two were in a school system, and one was conducted at home via telerehabilitation.

**Table 1:** Summary of included articles investigating virtual reality interventions to improve motor performance in neurologic populations.

**References**	**Study design**	**N (VR/CTL)**	**Population**	**Mean age (years)**
Bang et al., 2016	Randomized, repeated measures design	20/20	Stroke	62.7
Biffi et al., 2017	Single-group, repeated measures design	12	Acquired Brain Injury	12.1
Bonney et al., 2017	Randomized, repeated measures design; VR and Task-oriented Functional Training groups (TFT)	21/22	Developmental Coordination Disorder	14.3
Brien and Sveistrup, 2011	Single-subject, repeated measures multiple baseline design	4	Cerebral Palsy	16
Brütsch et al., 2011	Between groups, repeated measures design	10/14	Cerebral palsy, Spina Bifida, Traumatic Brain Injury, Lupus	12.1
Calabrò et al., 2017	Single-blind randomized controlled trial; Two groups—Robot-assisted gait training with VR and without VR.	20/20	Multiple Sclerosis	42.5
Cho et al., 2015	Randomized, repeated measures design	11/11	Chronic Stroke	59.3
Cho et al., 2016	Randomized, repeated measures design	9/9	Cerebral Palsy	9.8
Gandolfi et al., 2017	Multicenter, single-blind, randomized controlled trial	38/38	Parkinson's Disease	68.6
In et al., 2016	Randomized controlled trial	13/12	Chronic Stroke	55.9
Joong et al., 2009	Double-blinded randomized controlled trial; Two groups—VR vs. conventional physical therapy	12/12	Chronic Hemiparetic stroke	51.9
Killane et al., 2015	Between groups, repeated measures; Two groups—Freezing of Gait vs. Non-Freezing of Gait	13/7	Parkinson's Disease	64.1
Lamontagne et al., 2007	Between groups, repeated measures; Two groups—healthy vs. stroke	12/12	Stroke	66.0
Lee et al., 2015	Randomized, between groups, repeated measures design; VR vs. non-VR	10/10	Stroke	55.0
Liao et al., 2015	Single-blinded, stratified, randomized controlled trial. Stratified based on Hoehn and Yahr stage. Three groups—VR, Traditional therapy, Control	12/12/12	Parkinson's Disease	65.6
McEwen et al., 2014	Single-blinded, randomized controlled trial; Two groups—VR with dynamic weight-shifting vs. VR without dynamic weight-shifting	30/29	Stroke	64.1
Mirelman et al., 2009	Single-blinded, randomized clinical trial	9/9	Stroke	61.4
Mirelman et al., 2010	Single-blinded randomized controlled trial; Two groups—VR vs. Non-VR	9/9	Hemiparetic Stroke	62
Mirelman et al., 2011	Single group, repeated measures design	20	Parkinson's Disease	67.1
Park et al., 2013	Single-blinded, randomized controlled trial	8/8	Stroke	47.5
Pedreira da Fonseca, 2013	Sling-blinded, randomized clinical trial	14/13	Stroke	52.4
Peruzzi et al., 2017	Single-blind randomized controlled trial; Two groups—VR vs. non-VR	14/11	Multiple Sclerosis	42.8
Salem et al., 2012	Single-blinded randomized controlled trial	20/20	Developmental Delay	4.1
Schuler et al., 2011	Randomized control design; Two groups—Motor impairment vs. healthy in 2 training schedules	9/8	Cerebral Palsy, Brain Injury, Transverse Myelitis, Multiple Sclerosis	13
Singh et al., 2013	Multi-center controlled trial	15/13	Stroke	66.2
Song and Park, 2015	Randomized, repeated measure design; Two groups—VR vs. Non-VR	20/20	Stroke	57.7
van Gelder et al., 2017	Between groups, repeated measures; Two groups— CP vs. Healthy	16/11	Cerebral Palsy	10.5
Villiger et al., 2013	Single-group, repeated measures design	14	Incomplete Spinal Cord Injury	52.7
Villiger et al., 2017	Single-group, repeated measures design	12	Incomplete Spinal Cord Injury	57.5
Wall et al., 2015	Single-group, interrupted time series design	5	Incomplete Spinal Cord Injury	58.6
Yang et al., 2008	Single blind randomized controlled trial; VR vs. Conventional Exercise	11/9	Stroke	58.1
Yom et al., 2015	Randomized, repeated measures design; Two groups—VR vs. Non-VR	10/10	Stroke	71.4

#### Virtual Reality Interventions

Five of the 32 studies used an immersive form of VR. This was accomplished through either semi-cylindrical, multi-screen projection, or head mounted devices (HMDs). Twenty-seven of the 32 studies used a less-immersive VR intervention through use of 2D projection, IREX, RARS, Biorescue, YouRehab, WiiFit, or Xbox Kinect systems. A depiction of the VR systems and interventions of each study can be seen in Table 2.

**Table 2:** Summary of virtual reality interventions and findings of the included articles investigating virtual reality interventions on motor performance in neurologic populations.

**References**	**VR system**	**Virtual rehabilitation intervention**	**Findings**
Bang et al., 2016	Nintendo Wii Fit	40 min., 3 × /week, 8 weeks. Performed yoga, muscular strength exercise, aerobic exercise, and balancing exercise for 10 min. each	Significant improvement in balance for both groups following exercise program; significant between group differences. VR group demonstrated significant differences for walking (affected limb stance and swing phase, cadence) in post-testing.
Biffi et al., 2017	GRAIL system	10, 30 min. sessions over 3 weeks. Performed exercises belonging to 6 groups; load transfer, monopodalic load, walking, and endurance, dynamic balance, dynamic balance joint range of motion, motor coordination.	Significant improvement in gross motor abilities (standing and walking), endurance, and autonomy in functional activities. Significant decrease in the Gillette Gait Index on impaired side, and improved symmetry.
Bonney et al., 2017	Nintendo Wii	45 min., 1 × /week, 14 weeks. Played 8 games per session with progressive loading of training variables.	Significant improvement in muscular strength, motor proficiency, agility, and self-efficacy in both groups. No significant difference between groups.
Brien and Sveistrup, 2011	Interactive Rehabilitation and Exercise system (IREX)	2, 45 min. sessions, 30 min. rest, 5 consecutive days. Performed 3 consecutive sets of 5 IREX applications each session. Applications lasted 2 min., with 10 s. rest interval. Games consisted of soccer, snowboard, sharkbait, zebra crossing, and gravball.	Significant improvement in balance and gait.
Brütsch et al., 2011	Lokomat with 42-inch screen	4 randomly assigned conditions; 3 (30sec) validation trials, 7 min. treatment trials; VR trials consisted of soccer and navigation.	Significant main effect for training condition. Improved effectiveness in initiating active participation in VR conditions.
Calabrò et al., 2017	Lokomat-Pro	40 min. of RAGT + VR, 5x/week, 8 weeks with interactive avatar in VE. Subjects required to pass obstacles or catch objects appearing on a trail.	Small effect size and non-significant differences between groups favoring RAGT+VR for Berg Balance Scale and TUG.
Cho et al., 2015.	Treadmill with VR environment projected onto a 1,800 x 1,900 mm screen	30 min., 5 × /week. Subjects walked on the treadmill and viewed the VE while performing cognitive tasks. VE included real community environment projections (crosswalk, garden, etc.). Cognitive tasks included memorization, math, and verbal tasks.	Improvement in walking function under both single and dual task conditions, as well as for VR and control groups. Greater improvements in function for the VR group under dual task condition compared to control.
Cho et al., 2016	Treadmill with 42-inch TV projection; Nintendo Wii jogging program.	30 min., 3 × /week, 8 weeks. Walked on treadmill with jogging program	Gait and balance improved following training for the VR group than control.
Gandolfi et al., 2017	Nintendo Wii; Web-camera attached to computer to provide telerehabilitation via Skype.	50 min., 3x/week, 7 weeks. Exercised on Wii only during ON state performing 10 exergames; table tilt, penguin slide, balance bubble, ski slalom, skateboarding, perfect 10, tilt city, snowball fight, rhythm parade, and birds-eye bulls-eye.	Significant difference between-group on balance for VR group, and significant time X group interaction for gait improvements in clinic group. Improvements in all outcomes measures for both groups except frequency of falls.
In et al., 2016	Virtual Reality Reflection Therapy (VRRT)	Both groups performed 30 min. of convention physical therapy. Then, VR group performed 30 min., 5 × /week, 4 weeks of VRRT intervention.	Significant improvements in static and dynamic standing balance, and gait speed in VR group compared to control.
Joong et al., 2009	Interactive Rehabilitation and Exercise System (IREX).	30 min., 4x/week, 3 weeks in addition to 40 minutes of conventional physical therapy. Intervention targeted a weight-shifting, balance and stepping tasks	Significant improvement in dynamic standing balance, gait speed, cadence, step time, step length and stride length in VR group. Correlation between improved dynamic balance and gait velocity.
Killane et al., 2015	VR maze game designed by DFKI, Germany. Fifty five inch screen, 1 meter in front of participant. Wii to produce navigation in the VE.	20 min., 8 session, 2 weeks. VR maze which participates navigated while stepping-in-place. Time constraints and cognitive dual-tasks were added.	Significant improvement in dual-tasking, stepping time, stepping rhythmicity. Improvement in FOG episodes. Improvements great in the FOG group vs. Non-FOG.
Lamontagne et al., 2007	Kaiser Optics ProView™ XL50 (HMD). VE powered by CAREN-2 system (Motek).	Single collection; 2 conditions while walking in a virtual hallway. 1) continuously changing optic flow sinusoidally from 0 – 2x participant's comfortable walking speed 2) optic flow changes at discrete, constant speeds 0.25-2 × participant's comfortable walking speed.	Overall, demonstrated negative correlation between gait speed and optic flow speed. This relationship was weaker in stroke subjects than healthy subjects.
Lee et al., 2015	BioRescue platform.	45 min., 3 × /week, 6 weeks. City walking, hot air balloon, and bubble activities were used.	Significant improvement in dynamic standing balance following intervention in VR group and compared to control.
Liao et al., 2015	Wii Fit	45 min., 2 × /week, 6 weeks. Interactive avatar, yoga, strength, and balance exercises. 10, 15, and 20 min., respectively.	Greater improvement in obstacle crossing velocity, crossing stride length, dynamic balance, SOT, TUG, FES-I and PDQ39 in VR group than control. VR group also demonstrated significant improvement in movement velocity of limits-of-stability test than TE training.
McEwen et al., 2014	Interactive Rehabilitation and Exercise System (IREX)	10–12, 20 min. sessions. Standing VR games which dynamic weight-shift such as soccer and snowboarding were used.	Both groups improved dynamic balance and gait speed at the minimal clinical important difference for each outcome measure post-training. Effect sizes favored experimental group.
Mirelman et al., 2009	Rutgers Ankle Rehabilitation System (RARS)	60 min., 3 × /week, 4 weeks. Seated ankle dorsiflexion, plantar flexion, inversion, eversion and combined motions were performed to navigate a boat or train through a VE with movable targets.	Improvements in gait speed and distanced walked in VR group. Significant improvements in distance and steps walked in community in VR group which were maintained 3-months post-intervention.
Mirelman et al., 2010	Rutgers Ankle Rehabilitation System (RARS)	60 min., 3 × /week, 4 weeks. Navigated a plane or boat through the VE which contained a series of targets using only dorsiflexion, plantar flexion, inversion and eversion, or a combination of these movements.	VR group demonstrated significantly larger increase in ankle power generation in push-off, greater change in ankle ROM post-training, and significant differences in knee ROM on affected side during stance and swing. No significant differences in kinematics or kinetics at hip.
Mirelman et al., 2011	Outdoor virtual scene projected on a screen	3 × /week., 6 weeks. Process multiple stimuli and make decisions about obstacle negotiation in 2 planes, with distracters such as changing light and moving objects, while walking on a treadmill.	Significant improvement in gait speed during usual walking, dual tasks, and over-ground obstacle negotiation. Dual-task variability and Trail Making Test times improved. Retention effects noted 1 month later.
Park et al., 2013	Head-mount device (HMD)	Both groups performed 60 min., 5 × /week, 4 weeks of conventional physical therapy. VR group performed additional 30 min. 3 × /week, 4 weeks of VR intervention. Three-stage program: (1) supine trunk stability and pelvic tilt, (2) sitting trunk stability and pelvic tilting, and selective movements between each, (3) lower extremity muscle strengthening, standing trunk stability.	Significant improvement in gait parameters (except cadence) in VR group post-intervention, and at 1-month retention. No significant change in control group. Significant improvement in stride length in VR group compared to control.
Pedreira da Fonseca et al., 2017	Nintendo Wii	2 × /week for 20 sessions of conventional physical therapy. VR group received 15 min of conventional physical therapy plus 45 min. VR intervention. Tennis, hula hoop, soccer and boxing were used.	Improvement in dynamic balance and a reduction in falls in both VR and control groups. Significant reduction of falls demonstrated in VR group. Significant change in dynamic balance in control group.
Peruzzi et al., 2017	VE generated with WorldViz and projected on a 27-inch screen.	45 min., 3 × /week, 6 weeks. Virtual tree-line trail, passing objects appearing on the trail.	Significant improvements in walking endurance, speed, cadence, stride length, and lower extremity ROM in both groups. VR group significantly improved balance. VR group improved significantly more than control group in hip ROM and hip power at terminal stance in post-training.
Salem et al., 2012	Nintendo Wii Sports and Nintendo Wii Fit	30 min., 2 × /week, 10 weeks. VR training used balance, strength and walking games; lunges, single leg stance, soccer, penguin slide, tightrope, running, hula hoop, etc.	Significant improvement in single-limb balance and grip strength in VR group vs. control. Demonstrated trend toward greater improvement in dynamic balance, gait parameters and gross motor function in VR group vs. control.
Schuler et al., 2011	Lokomat	14 min./session. Two types of VR games; soccer game with virtual opponents and a landscape with objects to collect.	EMG activity significantly higher in both groups during tasks with VR than normal walking conditions.
Singh et al., 2013	Nintendo Wii Fit and Xbox 360 Kinect	30 min., 2 × /week, 6 weeks. Performed Balance Bubble on Wii Fit and Rally Ball on Kinect. Participants who mastered Rally Ball progressed to Reflex Ridge.	Significant improvement in the VR group for dynamic balance, however non-significant between-group findings.
Song and Park, 2015	Xbox Kinect	30 min., 5 × /week, 8 weeks. Kinect sports, sports season 2, adventure and gunstringer were used.	Improvement in balance and weight bearing in hemiparetic side in both groups.
van Gelder et al., 2017	Gait Real-time Analysis Interactive Lab (GRAIL).	2 trials/2 min. each. Visual feedback regarding knee and hip extension by presenting a bar plot with vertically moving ball and a target to visualize the goal knee/hip extension.	All children, except one, demonstrated improvement in hip and/or knee extension during gait in response to real-time feedback. Peak hip extension and peak knee extension significantly improved.
Villiger et al., 2013	YouRehab, YouKicker system	45 min., 4–5 × /week, 4 weeks. Footbag, Hamster Splash, Stark Kick, and Planet Drive were used.	Significant improvements in gait parameters, balance and strength of lower limbs following treatment and maintained 12–16 weeks after training.
Villiger et al., 2017	YouRehab, YouKicker system	30-45 min., 16-20 session, 4 weeks. Footbag, Planet Drive, Star Kick, Hamster Splash were used.	Significant improvements in lower limb strength, balance and functional mobility. Significant improvement in functional mobility maintained at 2–3-month retention.
Wall et al., 2015	Nintendo™ Wii Fit	60 min., 2 × /week, 7 weeks. Performed multiple games from Wii Fit to improve weight shifting, stability, balance and coordination.	Significant improvement in gait speed and dynamic balance following training, which were maintained 4-weeks post-training.
Yang et al., 2008	Visual screen, 3D acceleration graphics and 3D audio	20 min., 3x/week, 3 weeks. VE simulated a community in Taipei and was integrated to speed and incline changes of treadmill; scenarios included lane walking, street crossing, obstacle striding and park stroll.	VR group significantly improved walking speed, community walking times and WAQ score post-training and in 1 month retention. VR group significantly improved ABC which was not maintained at retention. VR group improved significantly more than controls.
Yom et al., 2015	Virtual reality-based ankle exercise (VRAE) using VRAE exercise program, computer and projector.	30 min., 5 × /week, 6 weeks.	Significant improvement in dynamic balance and muscle tone following VR intervention and compared to control group.

Eleven of the 32 studies used treadmill training in combination with their VR intervention. Three of these studies used robot-assisted gait training (RAGT), and eight used standard treadmill training (TT). One study used a balance platform which was integrated into their VR task. Two used the Rutgers ankle rehabilitation system (RARS). Three studies used the IREX VR system. Eight studies used commercial VR systems, Wii Fit (*n* = 7), or Kinect (*n* = 1), and one study used both the Nintendo Wii and Xbox Kinect.

All 32 studies reported improvements in the primary motor outcomes specific to each intervention with the use of VR. None of the studies compared the efficacy of immersive and non-immersive VR technologies. Additionally, none of the studies investigated the specific components of their intervention (i.e., adaptability, feedback, competition, etc.) to analyze the true effectiveness of each component.

#### Behavior Change Technique Coding

**Figure 3:**
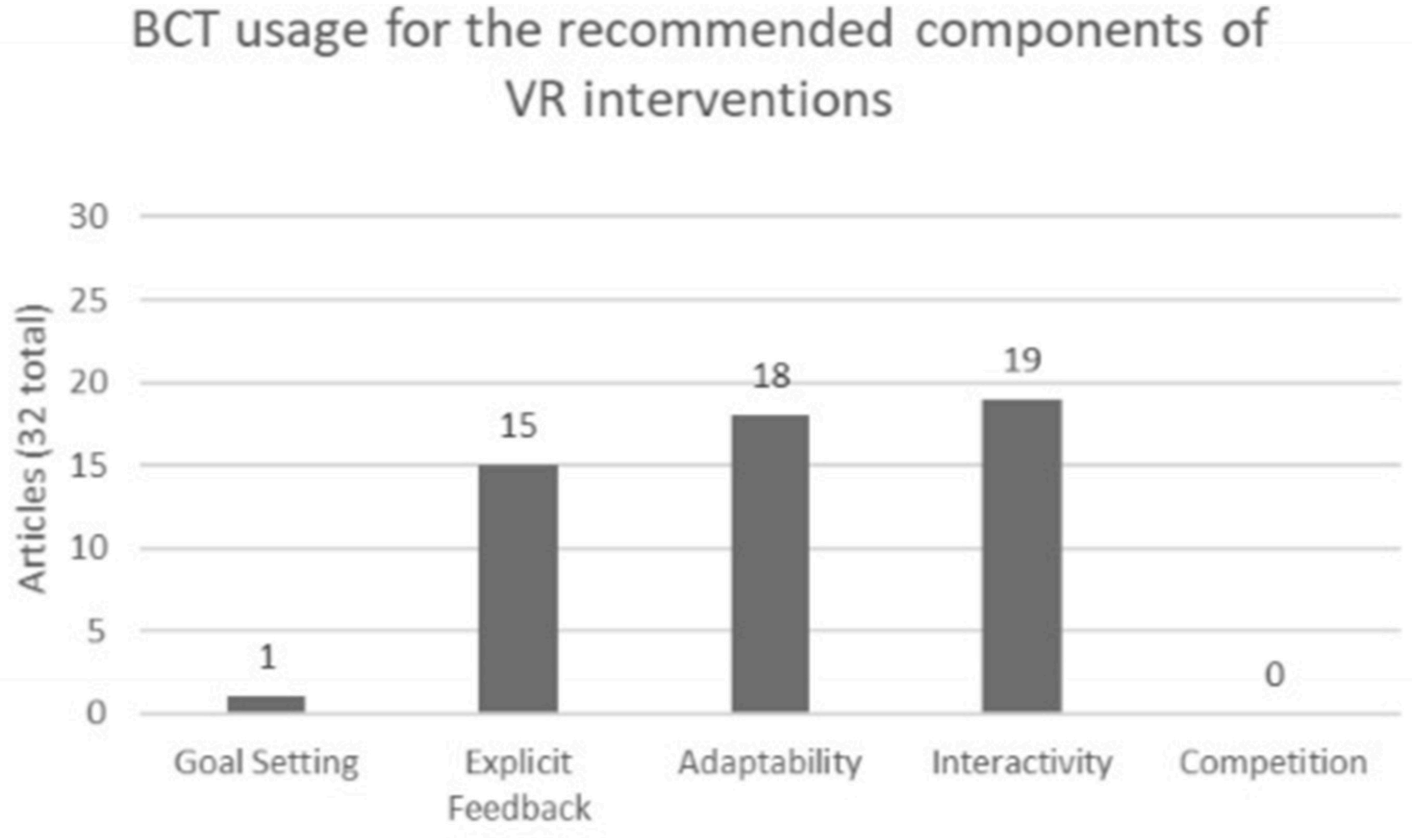
Summary of BCT usages in respect to the 5 recommended components of VR interventions across all 32 articles.

The number of studies that included at least one of the recommended BCT components are present in Figure 3. A breakdown of which components were included in each study is present in Table 3. There was substantial agreement (Cohen's Kappa = 0.714) for the initial thirty-percent of articles coded, improving to almost perfect agreement (Cohen's Kappa = 0.961) for the final seventy-percent of articles coded following the consensus meeting between the two coders. Eighteen studies (56.3%) used graded tasks (taxonomy code: 8.7) to adapt the VR intervention to optimally challenge the participants. For example, Villiger et al. (2013) graded task difficulty by adjusting the speed of the task or adding obstacles, which participants were instructed to avoid, in their VE. Fifteen studies (46.9%) provided explicit feedback to participants, six used feedback on performance of the behavior (taxonomy code: 2.2), and nine used feedback of outcomes on behavior (taxonomy code: 2.7). van Gelder et al. (2017) gave subjects feedback on performance of their target behavior through visual feedback provided via a bar plot and vertical ball which moved according to real-time joint angles in reference to a visual target. Liao et al. (2015) provided feedback on outcomes of behavior by providing subjects with their total score following conclusion of the VR task. The interactivity between the subjects' real-world movements and movements of the avatar in the VR is an example of biofeedback (taxonomy code: 2.6) which was used in nineteen (59.4%) of studies. One (3.1%) set explicit goals related to behavior outcome (taxonomy code:1.3) by explaining the goals of the VR training prior to beginning the protocol (Bonney et al., 2017). The option for adding competition to the VR task was not included in any study in this review. VR interventions which had a dynamic component to their VE were coded as “restructuring the physical environment” (taxonomy code: 12.1). Yang et al. (2008) accomplished this through by creating a VE with three-dimensional graphics and auditory outputs, which changed with the speed and incline of the treadmill used in the intervention. Five studies (15.6%) were coded as restructuring the physical environment.

Table 3Summary of behavior change techniques of included articles investigating virtual reality interventions to improve motor performance in neurologic populations.

**Table:** 

**BCT label**	**Bang et al. (2016)**	**Biffi et al. (2017)**	**Bonney et al. (2017)**	**Brien and Sveistrup (2011)**	**Brütsch et al. (2011)**	**Calabrò et al. (2017)**	**Cho et al. (2015)**	**Cho et al. (2016)**	**Gandolfi et al. (2017)**	**In et al. (2016)**	**Joong et al. (2009)**	**Killane et al. (2015)**	**Lamontagne et al. (2007)**	**Lee et al. (2015)**	**Liao et al. (2015)**
**1. Goals and planning**															
1.1 Goal setting (behavior)															
1.3 Goal setting (outcome)			X												
**2. Feedback and monitoring**															
2.1 Monitoring of behavior by others without feedback						X									
2.2 Feedback on behavior		X				X					X				X
2.6 Biofeedback	X	X		X	X	X	X	X	X		X	X			X
2.7 Feedback on outcome(s) of behavior											X				X
**4. Shaping**															
4.1 Instruction on how to perform the behavior					X	X			X						
**6. Comparison of behavior**															
6.1 Demonstration of behavior															
6.2 Social comparison															
**8. Repetition and substitution**															
8.1 Behavior practice	X	X	X	X		X	X	X	X	X	X	X		X	X
8.7 Graded task			X	X				X	X	X	X	X			
**12. Antecedents**															
12.1 Restructuring the physical environment						X						X			
**15. Self-belief**															
15.1 Verbal persuasion			X

**Table:** 

**BCT Label**	**McEwen et al. (****2014****)**	**Mirelman et al. (****2009****)**	**Mirelman et al. (****2011****)**	**Mirelman et al. (****2010****)**	**Park et al. (****2013****)**	**Pedreira da Fonseca et al. (****2017****)**	**Peruzzi et al. (****2017****)**	**Salem et al. (****2012****)**	**Schuler et al. (****2011****)**	**Singh et al. (****2013****)**	**Song and Park (2015)**	**van Gelder et al. (****2017****)**	**Villiger et al. (****2013****)**	**Villiger et al. (****2017****)**	**Wall et al. (****2015****)**	**Yang et al. (****2008****)**	**Yom et al. (****2015****)**
**1. Goals and planning**																	
1.1 Goal setting (behavior)																	
1.3 Goal setting (outcome)																	
**2. Feedback and monitoring**																	
2.1 Monitoring of behavior by others without feedback																	
2.2 Feedback on behavior		X										X					
2.6 Biofeedback		X		X			X		X		X	X	X	X			
2.7 Feedback on outcome(s) of behavior		X	X	X			X			X			X	X			
**4. Shaping**																	
4.1 Instruction on how to perform the behavior																	
**6. Comparison of behavior**																	
6.1 Demonstration of behavior																	X
6.2 Social comparison																	
**8. Repetition and substitution**																	
8.1 Behavior practice	X	X	X	X		X	X	X		X	X		X	X	X	X	X
8.7 Graded task		X	X	X			X	X	X	X		X	X	X		X	
**12. Antecedents**																	
12.1 Restructuring the physical environment			X				X									X	
**15. Self-belief**																	
15.1 Verbal persuasion									X								

## Discussion

### Summary of Main Findings

The purpose of this study was to systematically review the existing VR literature aimed at improving motor performance in neurologic populations and analyze the underlying BCTs by coding each intervention using the BCTTv1. Studies were included if they directly investigated the role of VR in improving gross motor performance in neurologic populations. Only the VR component of the intervention was considered during the BCT coding. Thirty-two studies were included in total.

Overall, it is clear that VR interventions for motor rehabilitation of neurologic populations is in its infancy. There is limited diversity in patient populations, most studies use adult participants, and many of the studies were performed in controlled environments. Post-stroke populations were the most commonly studied, used in 46.9% of the studies included in this review. Also, most studies used a non-immersive form of VR intervention. Moreover, none of the articles investigated the efficacy of individual components of their VR intervention to better understand the significance of the motor performance outcomes. All studies reported improvements in motor outcomes, however it is difficult to point to the most effective component if systematic investigation into the specific parts of a VR intervention is not performed, especially when trying to analyze the most effective active ingredients, or BCTs, of that intervention.

Improvements in technology have also increased the usage of telerehabilitation to enhance home-based physical rehabilitation (Latifi, 2008). There has been emerging investigation of the role of VR in telerehabilitation of the upper extremity as well as balance (Popescu et al., 2000; Golomb et al., 2010; Llorens et al., 2015). However, only one study investigated the use of VR in telerehabilitation of upright mobility (Gandolfi et al., 2017). Further investigation into the feasibility, efficacy and safety of VR interventions included in telerehabilitation protocols is needed.

Goal setting, explicit feedback, adaptability, interactivity, and competition have been identified as components of a successful VR task (Zimmerli et al., 2013). Given these recommended components, it was expected that codes would be used from the following main taxonomy areas: goals and planning, feedback and monitoring, comparison of behavior, and repetition and substitution. Goal setting and planning includes BCTs related to setting explicit task goals. These components were not readily used, as they were only incorporated in 1 of 32 studies (3.1%) of the articles in this review. Feedback and monitoring include BCTs related to providing feedback, monitoring behavior, and biofeedback. This section of the taxonomy applies to both the explicit feedback and interactivity components recommended for VR tasks, and at least one BCT from this area of the taxonomy was used in most of the studies included in this review (21 out of 32 studies; 65.6%). Fifteen studies utilized explicit feedback (46.9%) with studies providing either feedback on performance or feedback on outcomes of behavior. Biofeedback is defined in the BCTTv1 as a method of providing feedback about the body using an external monitoring device. Through use of interactive VR technologies, users can readily receive feedback from the VR. Therefore, interactivity of a VR task can be coded as using biofeedback, which was incorporated in 19 of 32 studies (59.4%) included in this review. Finally, the repetition and substitution section of the taxonomy includes “graded task,” which is used to set an easily attainable task that can gradually be increased in difficulty to achieve the desired outcome. This also speaks to the adaptability of a VR task. Of the recommended components of VR, adaptability was the most commonly adopted component, being incorporated into 18 out of 32 studies (56.3%) included in this review. These results illustrate the state of BCTs in VR tasks for neurorehabilitation. BCTs related to interactivity, feedback and adaptability of an intervention are represented well, however BCTs for explicit task goals and competition are lacking in the interventions included in this review. As suggested by Zimmerli et al. (2013), to create well-rounded VR interventions aimed to improve motor performance it is imperative to include multiple components into the VR task design. Therefore, interventions that fail to include aspects of goal setting, for example, may lose an important active ingredient to maximize the effectiveness of the VR intervention on neurorehabilitation and motor outcomes.

BCTs are often not deliberately stated in descriptions of behavioral interventions (Michie et al., 2009), and this holds true in the VR literature for motor rehabilitation. Thus, some aspects of the VR interventions that could be implied, but did not explicitly state the target behavior, could not be coded. Moreover, none of the included articles investigated individual components of their VR intervention. For this reason, it is difficult to identify the BCTs which provide the optimal augmentation of the VR intervention. However, interventions using graded tasks (taxonomy code: 8.7) and biofeedback (taxonomy code: 2.6) attributed the improved motor performance from the intervention to these components of the VR task. Graded tasks were used in 18 studies (56.3%), and biofeedback in 19 studies (59.4%). Twelve articles (37.5%) had VR tasks that used adaptability and interactivity together. A graded task is defined in the BCTTv1 as an intervention created to be initially easy to perform with increasing difficulty until the target behavior is accomplished. This speaks to the adaptability of the intervention. Biofeedback provides feedback about the state of the body using external monitoring. This suggests a level of interactivity in the VR task. This review suggests that these components improve patient engagement and motivation in the motor task (Schuler et al., 2011; Zimmerli et al., 2013) and should be considered for inclusion in future research.

As previously stated, only one article investigated the efficacy of individual components of their VR task. Zimmerli et al. found that adaptability leads to improvements in motor outcomes by allowing the VR task to be adapted to meet the individual needs of the user, and this produces the ideal psychophysiological state in which a participant is optimally stimulated, both mentally and physically (Zimmerli et al., 2013). Interactivity helps improve task-engagement, as well as optimizing rehabilitation potential and motor outcomes. Conversely, components of feedback frequency and goal setting were found to not produce a significant improvement in the motor performance of subjects with neurologic disorders.

In terms of neurologic rehabilitation, the goal of including tasks is to change behavior to improve motor outcomes. Behavior change is complex, requiring a combination of multiple components to produce the desired outcome (Michie et al., 2013). In this way, it is important to identify the BCTs currently used successfully in VR literature, as well as the shortcomings. In this review, coders looked for BCTs related to the specific recommended components of a VR intervention, as well as identify BCTs present in the VR task which were outside of these recommendations. In terms of the recommended VR components and their associated BCTs, feedback, adaptability and interactivity are the most widely used in the thirty-two articles reviewed. However, the remaining two components, goal setting and competition, were not incorporated well into the VR tasks included in this review. Outside the 5 recommended components, the most used BCT overall was behavioral practice (taxonomy code: 8.1); 84.4% of the studies created their VR intervention to rehearse the performance of the desired behavior which is important with regard to motor rehabilitation. Changing the environment to facilitate the desired behavior change is deemed restructuring the physical environment (12.1), this BCT was used in 15.6% of the studies. These studies created a dynamic component within their VR which was not present at the beginning of the task, restructuring the VE to influence the wanted behavior change.

From this review, it was observed that some BCTs are being integrated into VR tasks to meets the needs of the user and create meaningful changes in motor performance. These include feedback (behavior and outcomes), graded tasks, biofeedback and behavioral practice, and have been shown to change physical activity behavior (Duff et al., 2017; Samdal et al., 2017). Conversely, from the BCT literature, it has been shown that goal setting and self-monitoring is effective for creating behavior change in general (Michie et al., 2009). Self-monitoring of behavior was not used in any of the articles in this review, and goal setting was only incorporated into 3.1% of the studies in this review.

This review provides important information regarding the use of BCTs in the design of VR interventions to improve motor outcomes in neurologic clinic populations. Specifically, this review investigated the components of each VR intervention, itself, rather than the complete intervention protocol. Some BCTs are being incorporated well while others are not. However, more comprehensive VR tasks designed with deliberate integration and testing of BCTs are needed. This will allow for better identification of the true active ingredients promoting change within a successful VR task, which BCTs are best for VR interventions in motor rehabilitation and improve replicability of clinically meaningful VR interventions. Addressing these gaps will allow for more tailored treatments to be developed in the physical rehabilitation domain, similar to those currently being implemented in the cognitive rehabilitation domain (Freeman, 2008; Parsons and Rizzo, 2008; Li et al., 2011; Wang and Reid, 2011; Gonçalves et al., 2012; Opriş et al., 2012; Kandalaft et al., 2013). This will help spring-forward this line of research to more rapidly catch up with its cognitive rehabilitation counterparts to more fully take advantage of VR therapy across multiple domains.

### Limitations

There are two limitations of note in this review. First, there is not an established way to apply the BCTTv1 to VR interventions. Other articles performing BCT coding using the BCTTv1 have established a clear framework for applying BCT codes to various technological interventions. However, these are mostly regarding mobile phone applications (Direito et al., 2014, 2016; Lyons et al., 2014; Middelweerd et al., 2014; Yang et al., 2015). Additionally, while some BCTs could be implied, if the text did not explicitly state or link the target behavior to the target population, it could not be coded.

## Conclusion

The purpose of this study was to investigate the ways in which VR was being used in motor rehabilitation of neurologic populations, and systematically code these interventions using the BCTTv1 to analyze the active ingredients of each VR task. The literature suggests that VR is a useful modality to design and implement effective rehabilitation interventions. However, the state of BCTs in VR is mixed. The literature suggests that graded tasks (adaptability) and biofeedback (interactivity) are two of the most consistent elements of a VR intervention for motor rehabilitation. BCTs are not often deliberately reported in the literature, and therefore cannot be coded. Moreover, studies did not investigate the specific components of their VR tasks making it difficult to link BCTs to significant components of VR interventions.

Further research into the specific components of VR interventions along with purposeful implementation and reporting of BCTs will help improve understanding of the efficacy of VR as a motor rehabilitation tool. Incorporation of BCTS into VR interventions could create the ideal intervention to enhance motor outcomes. Future research could benefit from incorporating BCTs, and what is currently known about BCTs from the physical activity literature, into the design process of VR interventions to produce optimal rehabilitation potential. However, it is acknowledged that the BCT framework is rarely used in physical rehabilitation intervention, and questions remain about its applicability to help enhance current practice. Nevertheless, we believe the inclusion of BCTs *a priori* could help our field systematically understand the extent to which VR could add value to current clinical approaches.

## Author Contributions

All authors contributed to the concept and design of the systematic review. DF performed the literature search. DF and JM performed the coding of all included articles according to the BCTTv1. DF drafted the initial manuscript. All authors contributed to manuscript revision and approved the manuscript for submission.

## Conflict of Interest Statement

The authors declare that the research was conducted in the absence of any commercial or financial relationships that could be construed as a potential conflict of interest.
